# Complete remission in PD-L1 high expression advanced gastric cancer patient with PD-L1 immunotherapy and chemotherapy integration treatment strategy: a case report

**DOI:** 10.3389/fonc.2025.1507411

**Published:** 2025-05-27

**Authors:** Xiaopeng Yu, Wenwen Zhao, Qingqing Feng, Jin Tian, Lili Zhao, Jun Xiao, Hongmei Wei

**Affiliations:** ^1^ Qingdao Central Hospital, University of Health and Rehabilitation Sciences (Qingdao Central Hospital), Qingdao, China; ^2^ Department of Oncology, Qingdao Hiser Hospital Affiliated of Qingdao University (Qingdao Traditional Chinese Medicine Hospital), Qingdao, China

**Keywords:** advanced gastric cancer, PD-L1, immunotherapy, diarrhea, case report

## Abstract

Immune checkpoint inhibitors (ICIs) have become the standard of care in treating patients with human epidermal growth factor receptor 2(HER2) negative gastric cancer, revolutionizing the treatment landscape. The combination of ICIs and chemotherapy has shown improved treatment efficacy and prolonged survival compared to chemotherapy alone. Despite these benefits, this combined treatment is also linked to a higher incidence of adverse events. In this report, we present a case that demonstrates exceptional therapeutic efficacy but also severe adverse reactions. In HER2-negative gastric cancer, patients with higher programmed death-ligand1 (PD-L1) expression demonstrate improved clinical outcomes compared to those with lower PD-L1expression. This case study presents a 66-year-old male diagnosed with stage IV poorly differentiated gastric adenocarcinoma, characterized by hepatogastric ligament involvement, multiple peritoneal lymph node metastases, and extensive liver metastases. Initially treated with oxaliplatin plus docetaxel chemotherapy, the patient exhibited an inadequate response after two cycles. Subsequently, due to high PD-L1 expression, the treatment approach was modified to paclitaxel albumin-bound combined with oxaliplatin and Sintilimab. A PET-CT scan on June 5, 2023, confirmed complete remission in a patient with advanced gastric adenocarcinoma expressing high levels of PD-L1, who had received PD-L1-specific immune therapy in combination with chemotherapy. The patient developed frequent diarrhea three weeks after the final treatment on February 25, 2023, which was managed symptomatically. Tragically, the patient succumbed to electrolyte imbalance and shock caused by complications from the diarrhea in July 2023.

## 1 Introduction

Gastric cancer is a highly malignant cancer and ranks as the second leading cause of cancer-related deaths worldwide (1, 2). Recently, immune checkpoint blockade has become a promising strategy for cancer treatment, with monoclonal antibodies targeting programmed cell death protein 1 (PD- 1), PD-L1, and cytotoxic T-lymphocyte-associated protein 4 (CTLA-4). In the context of treating advanced HER2-negative gastric cancer, the combination of immune checkpoint inhibitors with chemotherapy has shown significant survival benefits in terms of overall survival (OS) and progression-free survival (PFS) (3). The Rationale 305 trial revealed that tislelizumab plus chemotherapy led to a notable improvement in OS (HR 0.74 [95% CI: 0.59-0.94], median OS 17.2 vs 12.6 months; one-sided P = 0.0056) with manageable safety (4). Similarly, the ORIENT- 16 trial demonstrated that the use of immune therapy alongside chemotherapy in patients with CPS≥5 gastric adenocarcinoma resulted in a significant enhancement in OS (median 18.4 vs 12.9 months; HR 0.660; 95% CI 0.505-0.864; P = 0.0023) (5). These trials have validated the viability of this treatment strategy. In this report, we present a case study of a 66-year-old male diagnosed with stage IV poorly differentiated adenocarcinoma of the stomach who underwent treatment with immune checkpoint inhibitors combined with chemotherapy and achieved complete remission. The most frequently observed immune-related adverse events (irAEs) include colitis, endocrinopathies, hepatitis, and pneumonitis (2).In this report, we document a case of a common immune-related adverse event that tragically resulted in fatal outcomes.

## 2 Case report

**Figure 1:**
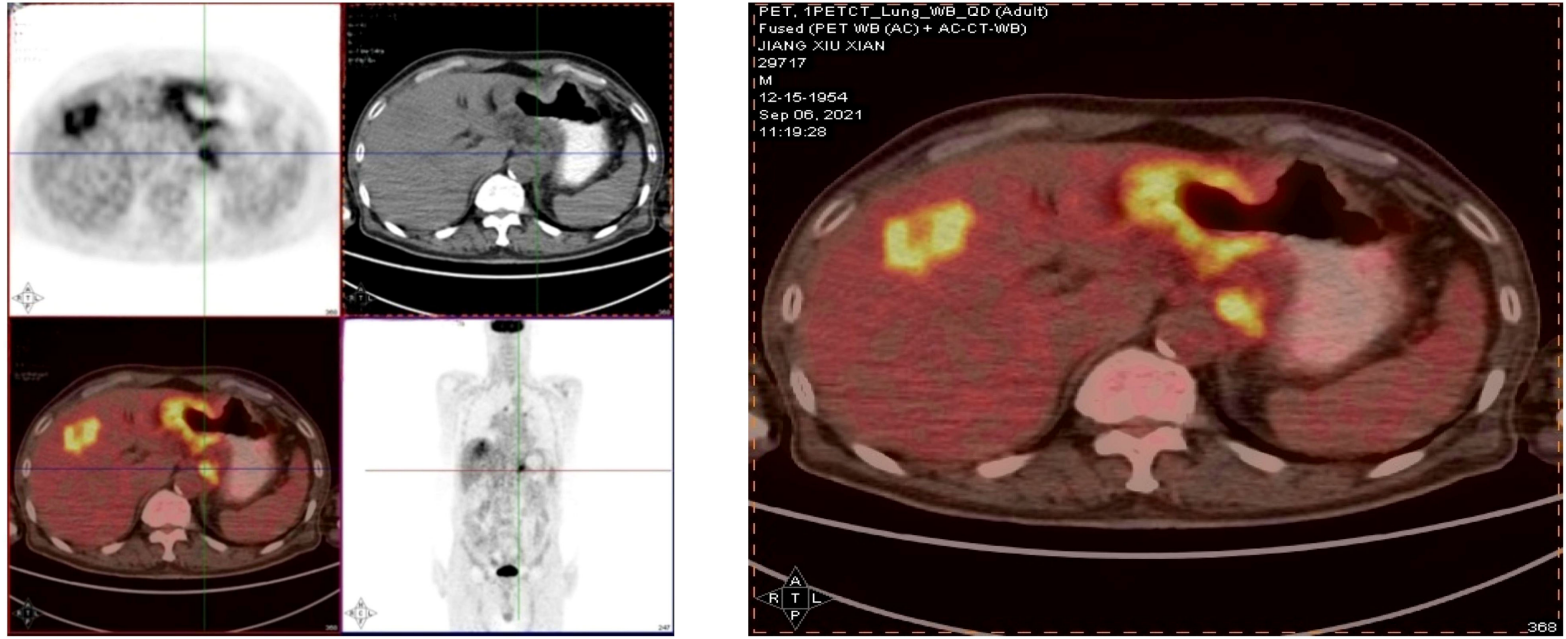
Suggestive of gastric cancer, infiltrating the serosal layer into the surrounding adipose tissue (peri-focal, hepatogastric ligament, retroperitoneal) lymph node metastasis, and liver metastasis.

Case description:A 66-year-old male presented in early August 2021 with unexplained weight loss, occasional heartburn, acid reflux, poor appetite, and fatigue. He had a 6-year history of diabetes, a smoking index of 1000 pack-years, and a 40-year history of consuming approximately 100ml of strong alcohol daily. The patient had no known drug allergies and no family history of tumors or genetic disorders. Upon admission, clear respiratory sounds were detected in the left lung during auscultation, while the right lung exhibited low respiratory sounds without dry or wet rales. Cardiac auscultation revealed no abnormalities, and there were no alpable or enlarged superficial lymph nodes. On September 3, 2021, the individual underwent enhanced computed tomography (CT) of the upper abdomen at a local hospital, revealing irregular thickening of the gastric wall along with multiple lymph node and liver metastases. Subsequent gastroscopy indicated irregular circumferential elevation, ulceration, and unclear boundaries affecting the entire gastric body, leading to gastric stenosis. Biopsy results confirmed poorly differentiated adenocarcinoma of the gastric body as well as Helicobacter pylori infection. Immunohistochemistry analysis revealed positive expressions of MLH1(+), MSH2(+), MSH6(+), and PMS2(+),indicating microsatellite stability. Additionally, PD-L1 (SP263) testing demonstrated 5% tumor cell (TC) expression, 10% immune cell (IC) expression, and a combined positive score (CPS) of 20. PET-CT confirmed infiltration of the gastric wall to the serosal layer with perigastric, liver gastric ligament, and retroperitoneal lymph node metastases, as well as multiple liver metastases (**Figure 1**). The patient did not receive any relevant antitumor therapy at an outside hospital. Referred to our hospital on September 8, 2021, for further diagnosis and treatment. Based on the laboratory tests and imaging, the patient was diagnosed with stage IV poorly differentiated adenocarcinoma of the stomach with perigastric, liver gastric ligament, and retroperitoneal lymph node metastases, as well as multiple liver metastases.

**Figure 2:**
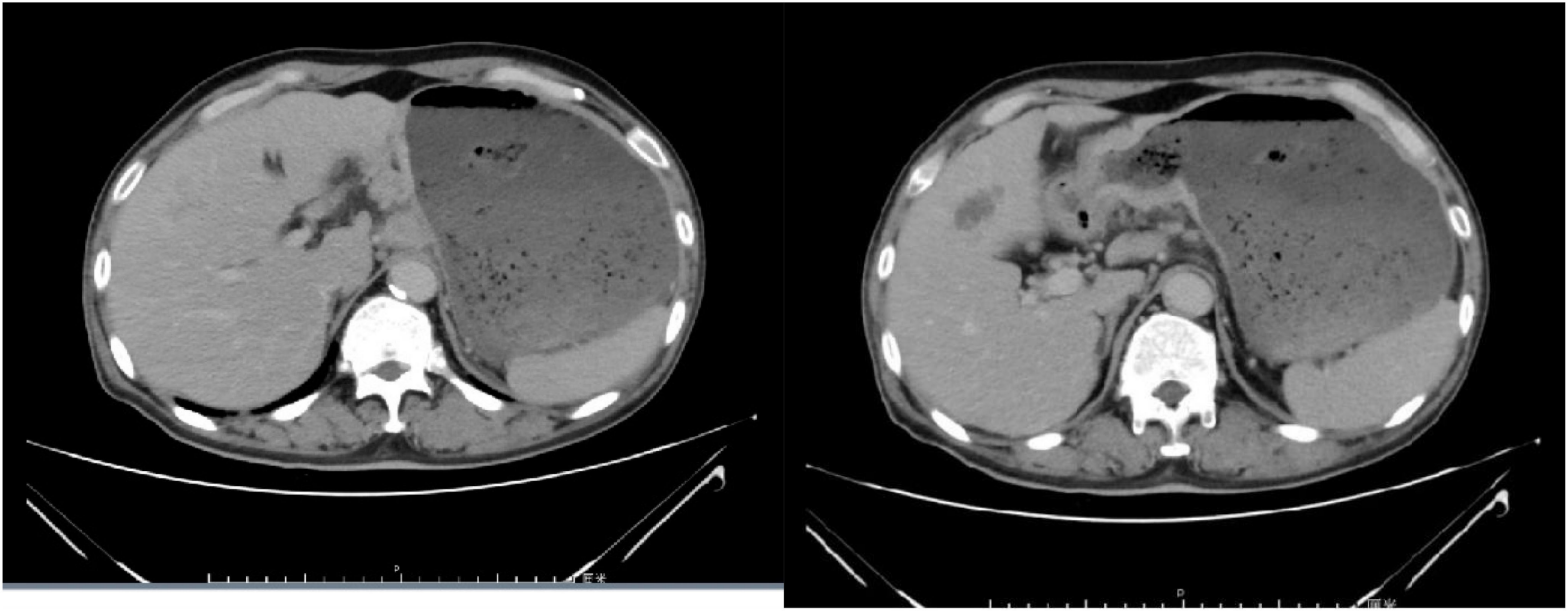
The efficacy evaluation after 2 cycles chemotherapy is Stable Disease (SD).

**Figure 3:**
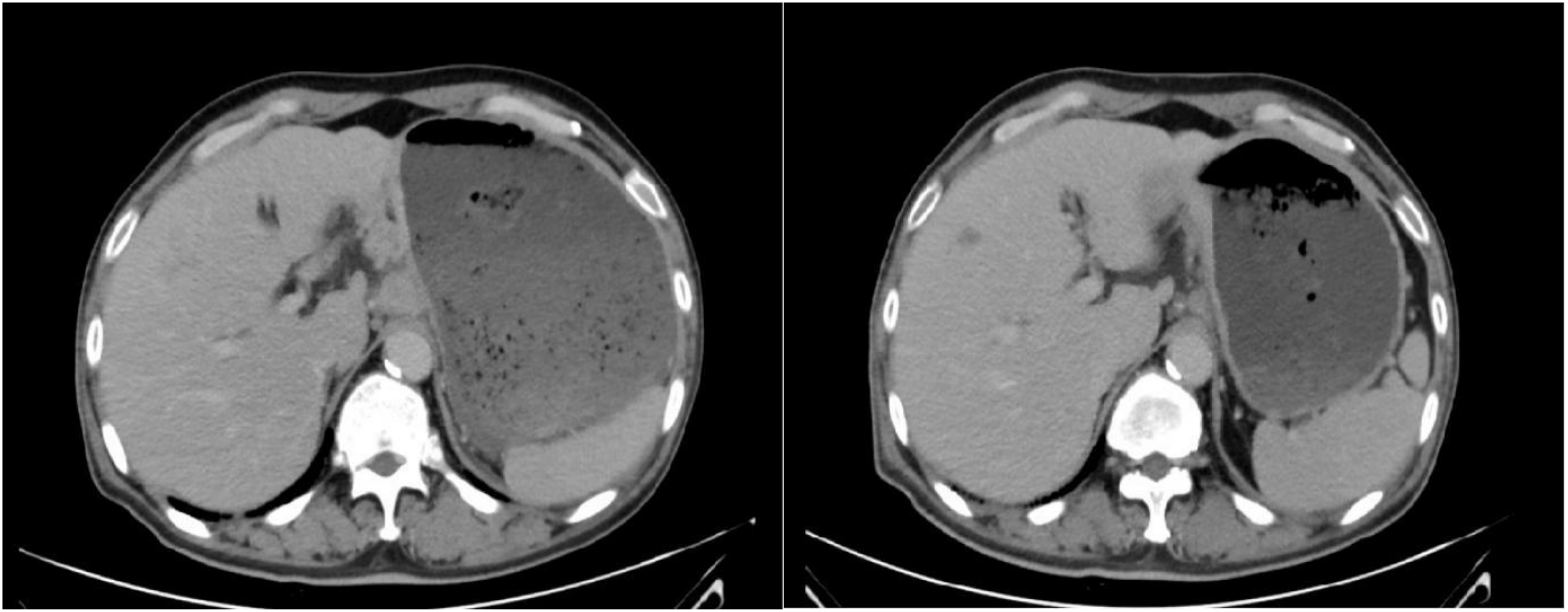
The efficacy evaluation after 2 cycles of immunotherapy combined with chemotherapy is Partial Response (PR).

**Figure 4:**
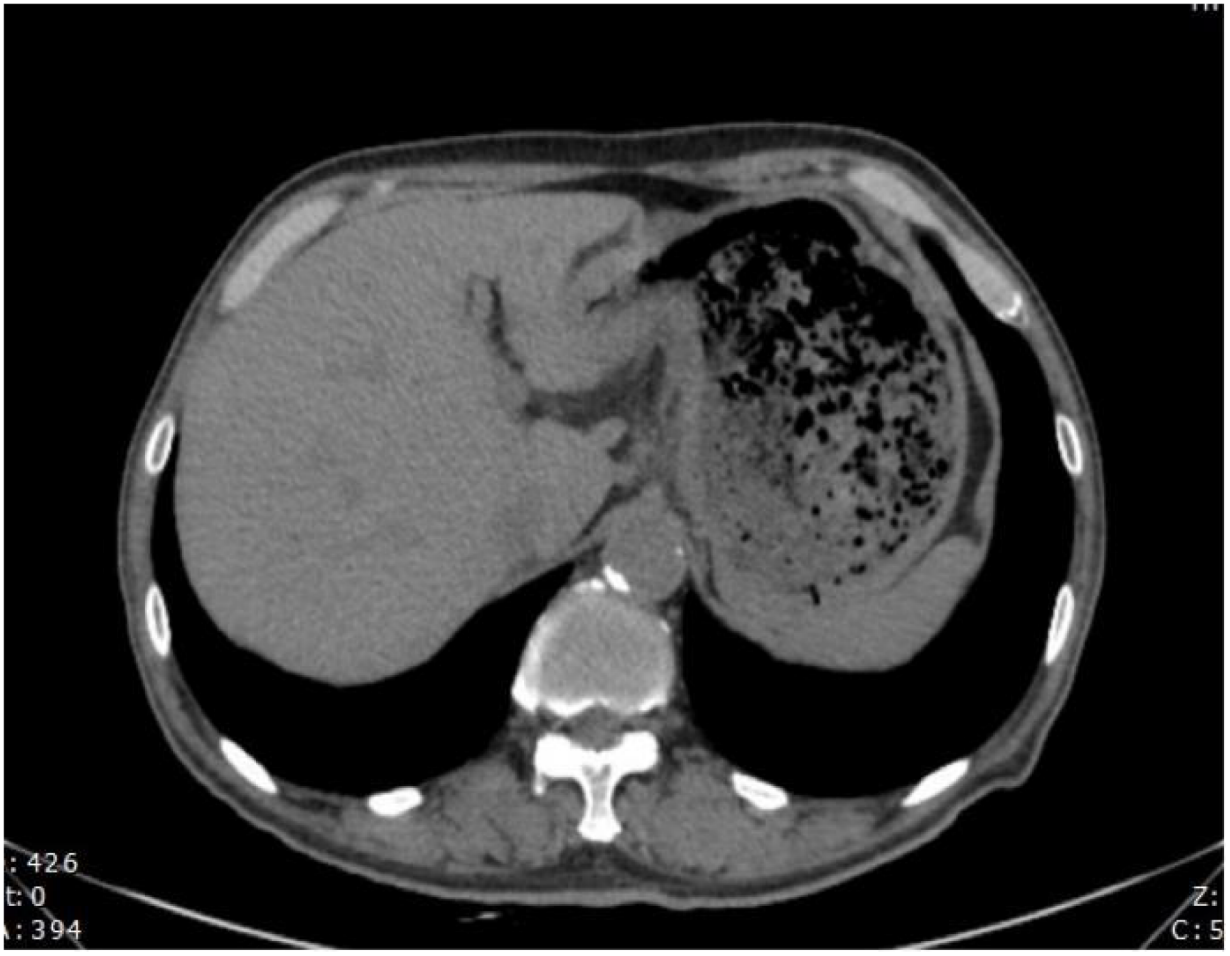
The efficacy evaluation after 4 cycles of immunotherapy combined with chemotherapy is Stable Disease (SD).

Chemotherapy with oxaliplatin and docetaxel was initiated on September 10, 2021, and continued for 2 cycles. Following two cycles, tumor markers did not decrease, and an enhanced computed tomography (CT) evaluation indicated stable disease (SD) (**Figure 2**). Due to the limited efficacy of chemotherapy alone and the patient’s PD-L1 CPS20 status, immunotherapy was incorporated into the treatment plan. A combination regimen of albumin-bound paclitaxel, cisplatin, and Sintilimab was administered on October 31, 2021; November 29, 2021; December 24, 2021; and January 18, 2022. The patient did not experience adverse reactions throughout the treatment, suggesting good safety. After four cycles, a partial response (PR) was observed on CT evaluation (**Figure 3**), followed by stable disease (SD) after six cycles (**Figure 4**). The maintenance plan included Sintilimab at a dose of 200mg every 21days, with the final treatment given on February 1, 2023.

**Figure 5:**
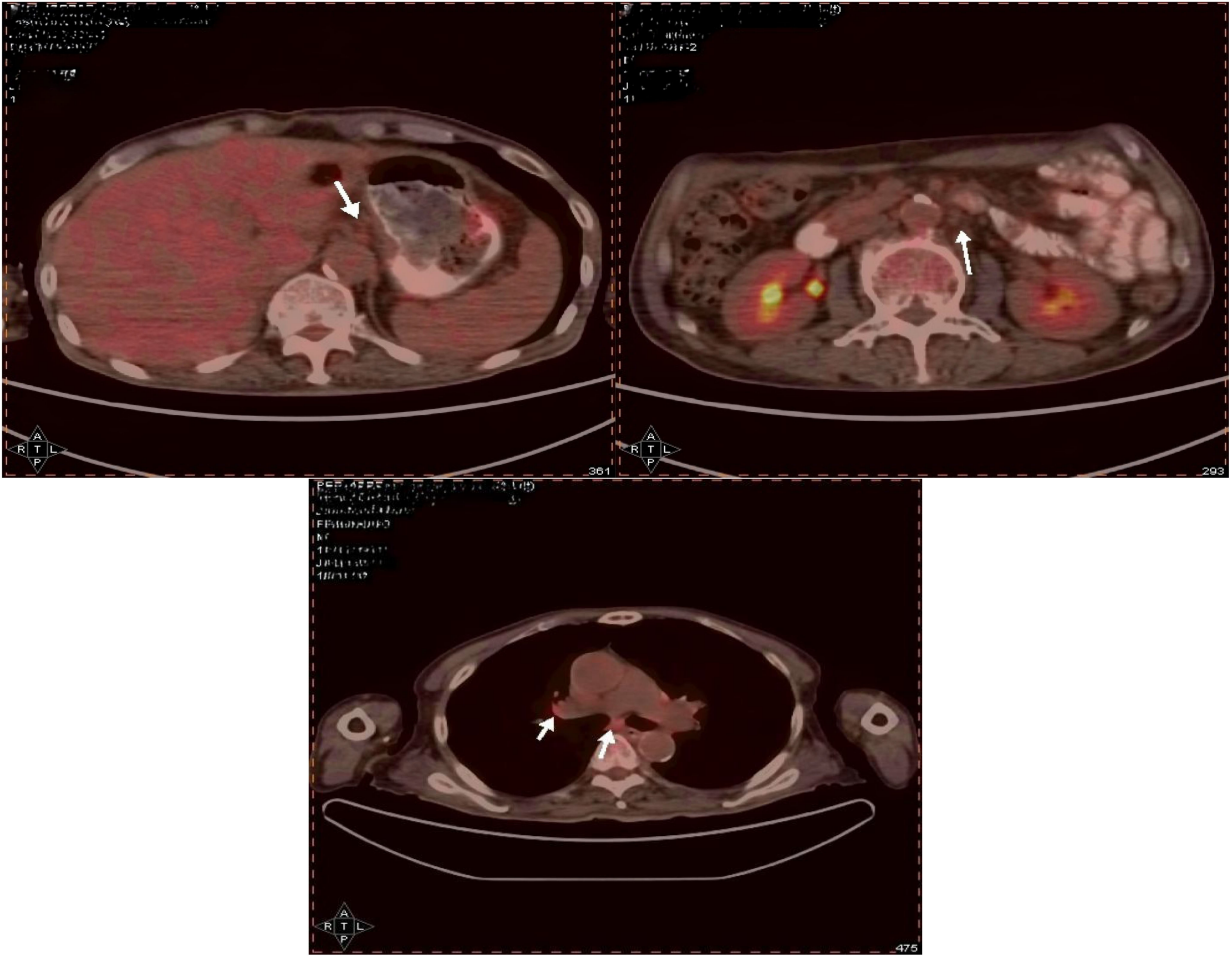
The gastric wall, liver, and lymph nodes show no increased FDG metabolism, suggesting that tumor activity is suppressed.

**Figure 6:**
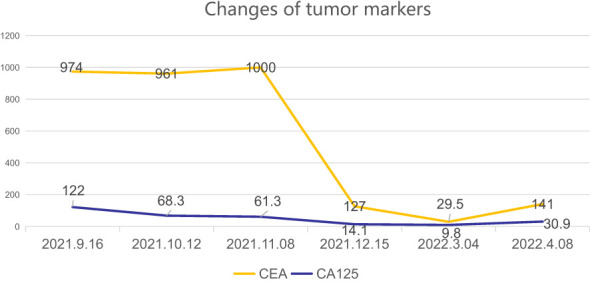
The levels of CEA (carcinoembryonic antigen) and CA- 125 (cancer antigen125) have shown significant decreases.

During the follow-up examination on June 5, 2023, at our hospital, a positron emission tomography-computed tomography (PET-CT) scan revealed mild thickening of the gastric wall and increased FDG metabolism, suggesting suppression of tumor activity (**Figure 5**). The tumor marker, carcinoembryonic antigen (CEA), was slightly elevated at 10.9 ng/mL, slightly above the normal range, while other markers remained within normal limits (**Figure 6**). Subsequent follow-up appointments were conducted every 3 months to monitor the patient’s progress. Imaging examinations consistently revealed no significant advancement of the disease, indicating a stable condition. As of the last evaluation (June 2023), the patient’s progression-free survival (PFS) has reached 21 months.

**Figure 7:**
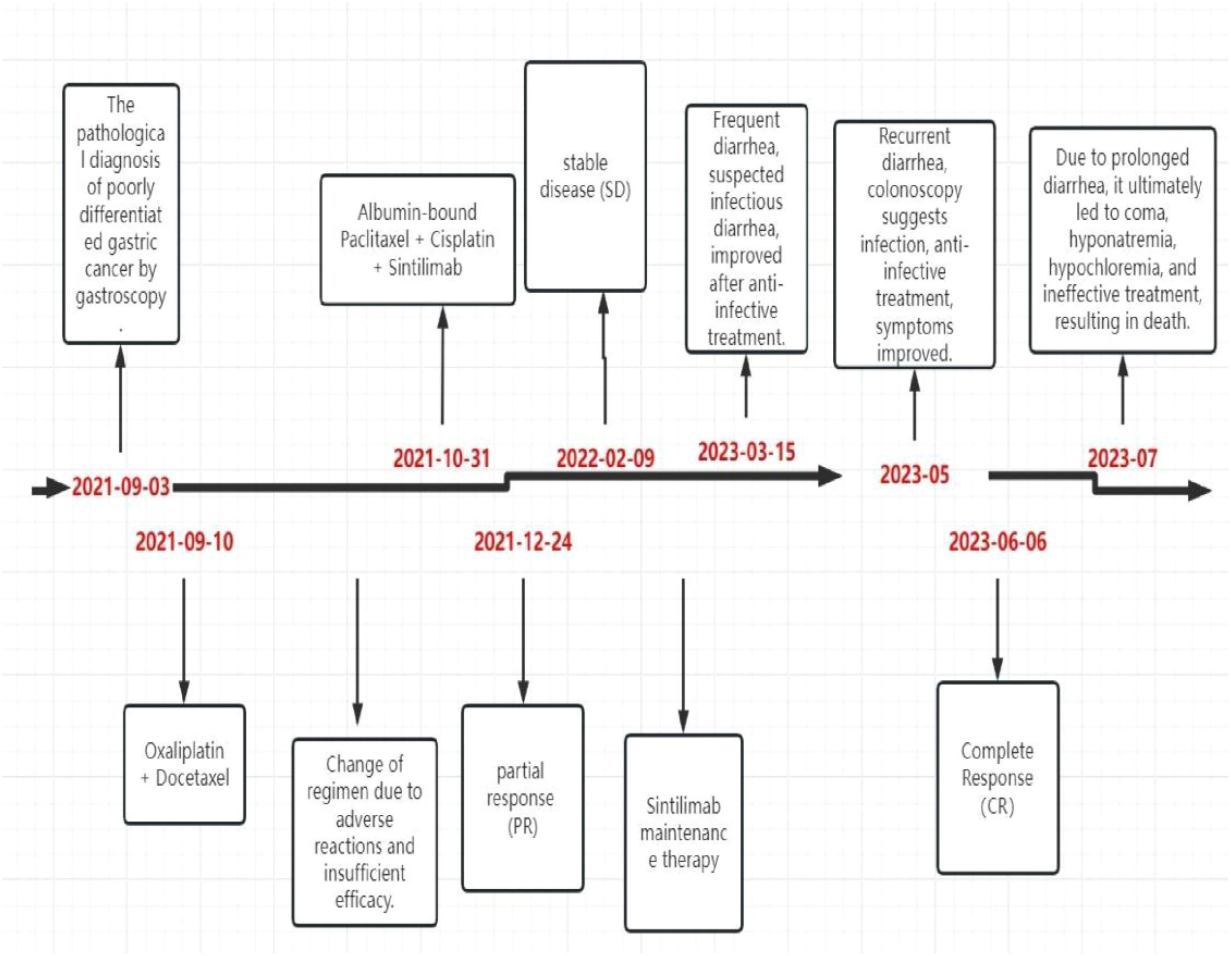
Treatment process and efficacy evaluation.

The patient did not experience any specific adverse reactions during treatment. Three weeks after the last treatment on February 25, 2023, the patient developed frequent diarrhea, passing loose stools 8–10 times daily, without abdominal pain, nausea, or vomiting. Symptoms improved after receiving treatment with steroids, gastric protectants, antibiotics, and hydration. However, the diarrhea recurred shortly thereafter, occurring 8 times daily. Upon evaluation in the gastroenterology department, the patient was diagnosed with infectious diarrhea. Treatment with antibiotics and hydration for one week led to symptom relief and discharge. One month later, the patient returned for a follow-up visit and did not report a recurrence of diarrhea. In May 2023, the patient experienced diarrhea again and underwent colonoscopy at a local hospital. The colonoscopy revealed continuous and diffuse lesions extending retrogradely from the distal rectum. These lesions were characterized by rough granular mucosa, diffuse congestion and edema, blurred and disappeared vascular pattern, fragile mucosa, bleeding, and severe areas with erosions or multiple shallow ulcers with purulent material adhering. Pathological findings indicated infiltration of inflammatory cells. Symptoms improved after symptomatic treatment, although specific medications were not specified. During a follow-up on June 4, 2023, the patient did not report diarrhea or abdominal pain, and all auxiliary examinations were normal. However, in July 2023, the patient sought care at another hospital due to persistent diarrhea, which led to coma, hyponatremia, hypochloremia, ineffective treatment, and eventually death. The timetable for diagnosis and treatment intervention for patients is as follows. (**Figure 7**).

## 3 Discussion

In China, 80% of gastric cancer patients are diagnosed at an advanced stage, leading to missed opportunities for surgical resection. HER-2 positive gastric cancer offers more treatment options compared to HER-2 negative cases. The KEYNOTE-811 trial showed a higher objective response rate of 74.4% with pembrolizumab versus 51.9% with placebo (P = 0.00006), including a complete response rate of 11.3% (6). However, only 10-22% of cases are HER-2 positive, with a larger proportion being HER-2 negative. For advanced HER-2 negative gastric cancer, first-line treatment options typically involve systemic chemotherapy or a combination of chemotherapy with anti-PD-L1/PD- 1 monoclonal antibodies (7). The CheckMate-649 trial revealed that the combination of nivolumab with chemotherapy led to a significant improvement in overall survival (OS) and progression-free survival (PFS) compared to chemotherapy alone, especially in patients with a Combined Positive Score (CPS) of 53 or higher. Most trials have indicated that immune combination therapy can be beneficial for patients regardless of PD-L1expression levels. Nevertheless, these studies have also highlighted that immune combination therapy tends to be more effective in patients with high PD-L1expression (3–5). Research has shown that patients treated with programmed cell death protein 1 (PD- 1) inhibitors have a 12.0% to 13.7% incidence of irAEs, with colitis occurring at a rate of 0.7% to 1.6%. The mechanisms behind irAEs are not fully understood, but it is believed to involve nonspecific T cell overactivation and increased cytokine expression, which can lead to a sepsis-like syndrome or abnormal activation of autoreactive T cells (1, 8). In the ORIENT- 16 study, the most common adverse events (incidence ≥ 30%) included decreased platelet count, decreased neutrophil count, decreased white blood cell count, anemia, nausea, vomiting, and increased aspartate aminotransferase.

Gastrointestinal toxicity from PD- 1/PD-L1 inhibitors typically occurred 6 to 8 weeks after starting therapy, but this patient experienced gastrointestinal adverse reactions only after 16 months of immunotherapy (5). Fortunately, this patient exhibits high PD-L1 expression, leading to the adoption of sintilimab in combination with albumin-bound paclitaxel and cisplatin. Subsequent evaluation revealed a complete response. The management of gastric adenocarcinoma poses ongoing challenges, prompting the exploration of novel therapeutic approaches. The integration of immune checkpoint inhibitors with chemotherapy emerges as a promising option for treating advanced HER-2 negative gastric cancer. Noteworthy results from Rationale 305, ORIENT- 16, and CheckMate 649 trials support the efficacy of combining immunotherapy with chemotherapy in patients with complete HER-2 negativity (3–5). These findings underscore the potential of anti-PD-L1 therapy in conjunction with chemotherapy for advanced HER-2 negative gastric cancer. Treatment selection for malignancies involves a dynamic process of refining strategies based on clinical outcomes. The favorable response observed in this case reinforces our confidence in the utility of anti-PD-L1 therapy combined with chemotherapy for advanced HER-2 negative gastric cancer. Collaborative efforts among researchers are anticipated to further enhance the efficacy of this treatment approach in the future.

Regrettably, the patient experienced severe adverse reactions that ultimately led to fatal consequences. Treated with PD- 1 inhibitors for gastric cancer, the patient developed diarrhea, with pathological findings indicating infiltration of inflammatory cells. Based on the patient’s clinical course, age at onset, pre-existing medication history, and symptoms post-drug treatment, a diagnosis of immune-related diarrhea was made. The patient’s demise was not attributed to the cancer itself, but rather to adverse reactions caused by the treatment, a somber realization. Despite showing excellent results of complete remission in the last PET-CT scan, the patient passed away a month later due to uncontrollable gastrointestinal adverse reactions. Had these reactions been manageable, it is possible that the patient’s survival could have been prolonged.

ICIs are a promising cancer treatment, but their use can lead to irAEs, which may affect multiple organs, ranging from mild side effects to severe, life-threatening complications. Currently, reliable biomarkers to predict the risk of immune-related adverse events in patients receiving immune checkpoint inhibitors are lacking. However, research on predictive biomarkers for ICI-related toxicity is progressing rapidly (9).

Common autoantibodies, such as antinuclear antibodies (ANA) and rheumatoid factor (RF), as well as organ-specific antibodies like anti-thyroid antibodies, are considered to have predictive potential (10–13). Additionally, cytokine profiles, such as tumor necrosis factor-α (TNF-α) (14), interleukin-6 (IL-6) (15), interleukin-17 (IL-17) (16, 17), and interleukin-10 (IL-10^)^ (18), may play a role in predicting irAEs. Proteins in serum and other bodily fluids have also shown predictive value for irAEs. Thyroid-stimulating hormone (TSH) is considered the most effective biomarker for monitoring thyroid dysfunction in patients treated with immune checkpoint inhibitors (19–21). Similarly, continuous measurements of serum B-type natriuretic peptide (BNP), troponin, and new-onset electrocardiogram abnormalities help predict cardiovascular irAEs (22). Fecal lactoferrin and calprotectin are commonly used to screen for immune checkpoint inhibitor-induced colitis (23), with calprotectin being particularly useful for assessing treatment response and reducing the need for repeat endoscopies (24).In addition to these biomarkers, baseline absolute counts of neutrophils, lymphocytes, monocytes, eosinophils, and basophils, platelet counts, and changes in white blood cells, lymphocytes, and eosinophils during follow-up are associated with an increased risk of immune-related adverse events. These methods are easy to implement in clinical practice, relatively low-cost, but most studies are retrospective analyses (25–32).

Moreover, research on biomarkers such as genetic variations, gene expression profiles, Human Leucocyte Antigen genotyping, micro RNAs, and gastrointestinal microbiota is gradually progressing (33–40). Despite significant advances in the study of potential predictive biomarkers for immune-related adverse events, no biomarker has been fully validated for widespread clinical application. Apart from routine laboratory tests, other biomarkers have not been recommended for analysis prior to immune checkpoint inhibitor treatment.

The increasing use of ICIs in oncology highlights the importance of monitoring patients for significant irAEs. Vigilance and thorough examination are crucial as irAEs are frequently diagnosed through exclusion. A multidisciplinary approach, involving input from consultants, is usually required to reach the correct diagnosis. Early initiation of glucocorticoids upon diagnosis is essential to prevent rapid deterioration and decrease the chances of severe, fatal outcomes.

## Data Availability

The original contributions presented in the study are included in the article/supplementary material. Further inquiries can be directed to the corresponding authors.

## References

[1] World Health Organization International Agency for Research on Cancer. GLOBOCAN 2018: estimated cancer incidence, mortality and prevalence worldwide. (Accessed June 16, 2020).

[2] The American Cancer Society. Cancer statistics center. (Accessed June 16, 2020).

[3] Janjigian YY Shitara K Moehler M Garrido M Salman P Shen L. First-line nivolumab plus chemotherapy versus chemotherapy alone for advanced gastric, gastro-oesophageal junction, and oesophageal adenocarcinoma (CheckMate 649): a randomised,open-label, phase 3 trial. Lancet. (2021) 398:27–40. doi: 10.1016/S0140-6736(21)00797-2 34102137 PMC8436782

[4] Moehler MH Kato K Arkenau H-T Oh D-Y Tabernero J Cruz-Correa M. Rationale 305: Phase 3 study of tislelizumab + chemotherapy vs placebo + chemotherapy as first-line treatment of advanced gastric or gastroesophageal junction adenocarcinoma. J ClinOncol. (2023) suppl 4):abstr 286. doi: 10.1200/JCO.2023.41.4_suppl.286

[5] Xu J Jiang H Pan Y Gu K Cang S Han L. LBA53 Sintilimab plus chemotherapy (chemo) versus chemo as first-line treatment for advanced gastric or gastroesophageal junction (G/GEJ) adenocarcinoma (ORIENT- 16): First results of a randomized, double-blind,phase III study. Ann Oncol. (2021) 32:S1331. doi: 10.1016/j.annonc.2021.08.2133

[6] Janjigian YY Kawazoe A Yañez P YañLi N YañLonardi S Kolesnik O. The KEYNOTE-811 trial of dual PD- 1 and HER2 blockade in HER2-positive gastric cancer. Nature. (2021) 600:727–30. doi: 10.1038/s41586-021-04161-3 PMC8959470 34912120

[7] National Comprehensive Cancer Network. NCCN clinical practice guidelinesin oncology: gastricCancer(Version1.2023). [DB/OL]. http://www.nccn.orghttp://www.nccn.org.

[8] Kennedy LB Salama AK. A review of cancer immunotherapy toxicity. CA Cancer J Clin. (2020) 70:86–104. doi: 10.3322/caac.21596 31944278

[9] Les I Martínez M Pérez-Francisco I Cabero M Teijeira L Arrazubi V. Predictive biomarkers for checkpoint inhibitor immune-related adverse events. Cancers. (2023) 15:1629. doi: 10.3390/cancers15051629 36900420 PMC10000735

[10] Kobayashi T Iwama S Yasuda Y Okada N Tsunekawa T Onoue T. Patients with antithyroid antibodies are prone to develop destructive thyroiditis by nivolumab: A prospective study. J Endocr Soc. (2018) 2:241–51. doi: 10.1210/js.2017-00432 PMC5836529 29600292

[11] Osorio JC Ni A Chaft JE Pollina R Kasler MK Stephens D. Antibody-mediated thyroid dysfunction during T-cell checkpoint blockade in patients with non-small-cell lung cancer. Ann Oncol. (2017) 28:583–9. doi: 10.1093/annonc/mdw640 PMC5834017 27998967

[12] Basak EA van der Meer JWM Hurkmans DP Schreurs MWJ Oomen-de Hoop E van der Veldt AAM. Overt thyroid dysfunction and anti-thyroid antibodies predict response to anti-PD-1 immunotherapy in cancer patients. Thyroid. (2020) 30:966–73. doi: 10.1089/thy.2019.0726 32151195

[13] Les I Martínez M Narro A Pérez I Sánchez C Puntí L. Association of immune-related adverse events induced by nivolumab with a battery of autoantibodies. Ann Med. (2021) 53:762–9. doi: 10.1080/07853890.2021.1931956 PMC8172225 34060971

[14] Luoma AM Suo S Williams HL Sharova T Sullivan K Manos M. Molecular pathways of colon inflammation induced by cancer immunotherapy. Cell. (2020) 182:655–671.e22. doi: 10.1016/j.cell.2020.06.001 32603654 PMC7415717

[15] Hunter CA Jones SA. Erratum: corrigendum: IL-6 as a keystone cytokine in health and disease. Nat Immunol. (2017) 18:1271. doi: 10.1038/ni1117-1271b 29044237

[16] McGeachy MJ Cua DJ Gaffen SL. The IL-17 family of cytokines in health and disease. Immunity. (2019) 50:892–906. doi: 10.1016/j.immuni.2019.03.021 30995505 PMC6474359

[17] Lo C-Y Wang C-H Wang C-W Chen C-J Huang H-Y Chung F-T. Increased interleukin-17 and glucocorticoid receptor-β Expression in interstitial lung diseases and corticosteroid insensitivity. Front Immunol. (2022) 13:905727. doi: 10.3389/fimmu.2022.905727 35865549 PMC9294725

[18] Saraiva M Vieira P O’Garra A. Biology and therapeutic potential of interleukin-10. J Exp Med. (2020) 217:e20190418. doi: 10.1084/jem.20190418 31611251 PMC7037253

[19] Muir CA Clifton-Bligh RJ Long GV Scolyer RA Lo SN Carlino MS. Thyroid immune-related adverse events following immune checkpoint inhibitor treatment. J Clin Endocrinol Metab. (2021) 106:e3704–13. doi: 10.1210/clinem/dgab263 33878162

[20] Yoon JH Hong AR Kim HK Kang H-C. Characteristics of immune-related thyroid adverse events in patients treated with PD-1/PD-L1 inhibitors. Endocrinol Metab. (2021) 36:413–23. doi: 10.3803/EnM.2020.906 PMC8090457 33820396

[21] Luongo C Morra R Gambale C Porcelli T Sessa F Matano E. Higher baseline TSH levels predict early hypothyroidism during cancer immunotherapy. J Endocrinol Investig. (2021) 44:1927–33. doi: 10.1007/s40618-021-01508-5 PMC8357750 33576954

[22] Isawa T Toi Y Sugawara S Taguri M Toyoda S. Incidence, clinical characteristics and predictors of cardiovascular immune-related adverse events associated with immune checkpoint inhibitors. Oncologist. (2022) 27:e410–9. doi: 10.1093/oncolo/oyac056 PMC9074992 35348766

[23] Gong Z Wang Y. Immune checkpoint inhibitor–mediated diarrhea and colitis: A clinical review. JCO Oncol Pract. (2020) 16:453–61. doi: 10.1200/OP.20.00002 32584703

[24] Zou F Wang X Glitza Oliva IC McQuade JL Wang J Zhang HC. Fecal calprotectin concentration to assess endoscopic and histologic remission in patients with cancer with immune-mediated diarrhea and colitis. J Immunother Cancer. (2021) 9:e002058. doi: 10.1136/jitc-2020-002058 33436487 PMC7805368

[25] Nakamura Y Tanaka R Maruyama H Ishitsuka Y Okiyama N Watanabe R. Correlation between blood cell count and outcome of melanoma patients treated with anti-PD-1 antibodies. Jpn J Clin Oncol. (2019) 49:431–7. doi: 10.1093/jjco/hyy201 30753621

[26] Ma Y Ma X Wang J Wu S Wang J Cao B. Absolute eosinophil count may be an optimal peripheral blood marker to identify the risk of immune-related adverse events in advanced Malignant tumors treated with PD-1/PD-L1 inhibitors: A retrospective analysis. World J Surg Oncol. (2022) 20:242. doi: 10.1186/s12957-022-02695-y 35897018 PMC9331074

[27] Ruste V Goldschmidt V Laparra A Messayke S Danlos F-X Romano-Martin P. The determinants of very severe immune-related adverse events associated with immune checkpoint inhibitors: A prospective study of the French REISAMIC registry. Eur J Cancer. (2021) 158:217–24. doi: 10.1016/j.ejca.2021.08.048 34627664

[28] Liu W Liu Y Ma F Sun B Wang Y Luo J. Peripheral blood markers associated with immune-related adverse effects in patients who had advanced non-small cell lung cancer treated with PD-1 inhibitors. Cancer Manage Res. (2021) 13:765–71. doi: 10.2147/CMAR.S293200 PMC7850423 33536784

[29] Fujimoto A Toyokawa G Koutake Y Kimura S Kawamata Y Fukuishi K. Association between pretreatment neutrophil-to-lymphocyte ratio and immune-related adverse events due to immune checkpoint inhibitors in patients with non-small cell lung cancer. Thorac Cancer. (2021) 12:2198–204. doi: 10.1111/1759-7714.14063 PMC8327687 34173724

[30] Michailidou D Khaki AR Morelli MP Diamantopoulos L Singh N Grivas P. Association of blood biomarkers and autoimmunity with immune related adverse events in patients with cancer treated with immune checkpoint inhibitors. Sci Rep. (2021) 11:9029. doi: 10.1038/s41598-021-88307-3 33907229 PMC8079370

[31] Xu H Feng H Zhang W Wei F Zhou L Liu L. Prediction of immune-related adverse events in non-small cell lung cancer patients treated with immune checkpoint inhibitors based on clinical and hematological markers: real-world evidence. Exp Cell Res. (2022) 416:113157. doi: 10.1016/j.yexcr.2022.113157 35427598

[32] Takada S Murooka H Tahatsu K Yanase M Umehara K Hashishita H. Identifying early predictive markers for immune-related adverse events in nivolumab-treated patients with renal cell carcinoma and gastric cancer. Asian Pac J Cancer Prev. (2022) 23:695–701. doi: 10.31557/APJCP.2022.23.2.695 35225483 PMC9272606

[33] Zhang Z Xie T Qi C Zhang X Shen L Peng Z. Peripheral blood biomarkers predictive of efficacy outcome and immune-related adverse events in advanced gastrointestinal cancers treated with checkpoint inhibitors. Cancers. (2022) 14:3736. doi: 10.3390/cancers14153736 35954401 PMC9367581

[34] Iafolla MAJ Yang C Chandran V Pintilie M Li Q Bedard PL. Predicting toxicity and response to pembrolizumab through germline genomic HLA class 1 analysis. JNCI Cancer Spectr. (2021) 5:pkaa115. doi: 10.1093/jncics/pkaa115 33554038 PMC7853183

[35] Berman D Parker SM Siegel J Chasalow SD Weber J Galbraith S. Ipilimumab efficacy and safety in patients with advanced melanoma: A retrospective analysis of HLA subtype from four trials. Cancer Immun. (2010) 10:6. doi: 10.1158/1424-9634 20957980 PMC2964017

[36] Marschner D Falk M Javorniczky NR Hanke-Müller K Rawluk J Schmitt-Graeff A. MicroRNA-146a regulates immune-related adverse events caused by immune checkpoint inhibitors. JCI Insight. (2020) 5:e132334. doi: 10.1172/jci.insight.132334 32125286 PMC7213806

[37] Hart M Nickl L Walch-Rueckheim B Krammes L Rheinheimer S Diener C. Wrinkle in the plan: miR-34a-5p impacts chemokine signaling by modulating CXCL10/CXCL11/CXCR3-axis in CD4, CD8 T cells and M1 macrophages. J Immunother Cancer. (2020) 8:e001617. doi: 10.1136/jitc-2020-001617 33229509 PMC7684812

[38] Sakurai T De Velasco MA Sakai K Nagai T Nishiyama H Hashimoto K. Integrative analysis of gut microbiome and host transcriptomes reveals associations between treatment outcomes and immunotherapy-induced colitis. Mol Oncol. (2022) 16:1493–507. doi: 10.1002/1878-0261.13062 PMC8978521 34270845

[39] Mao J Wang D Long J Yang X Lin J Song Y. Gut microbiome is associated with the clinical response to anti-PD-1 based immunotherapy in hepatobiliary cancers. J Immunother Cancer. (2021) 9:e003334. doi: 10.1136/jitc-2021-003334 34873013 PMC8650503

[40] Chau J Yadav M Liu B Furqan M Dai Q Shahi S. Prospective correlation between the patient microbiome with response to and development of immune-mediated adverse effects to immunotherapy in lung cancer. J Clin Oncol. (2021) 39:e21024. doi: 10.1186/s12885-021-08530-z PMC8278634 34256732

